# Exploring Personality Traits, Coping Strategies, and 5-Year Change in Blood Pressure in Young Adults: The African-PREDICT Study

**DOI:** 10.1177/23333928241271081

**Published:** 2024-11-25

**Authors:** Anchenique Papenfus, Karel Botha, Carina Mels, Marike Cockeran

**Affiliations:** 1 Subject Group Psychology, 56405 North-West University, Potchefstroom, South Africa; 2 Hypertension in Africa Research Team (HART), 56405 North-West University, Potchefstroom, South Africa; 3 Subject Group Statistics, 56405 North-West University, Potchefstroom, South Africa

**Keywords:** African-PREDICT study, blood pressure, cardiovascular disease, coping strategies, personality traits

## Abstract

This sub-study of the African Prospective Study on the Early Detection and Identification of Cardiovascular Disease and Hypertension (African-PREDICT) explored possible early psychological predictors of change in blood pressure. In a sample of normotensive at baseline black and white South Africans (*n *= 105; mean age at baseline 24.93), this study investigated the relationship between personality traits, coping strategies, and 24-hour ambulatory blood pressure measured at baseline and 5-year follow-up. Another aim was to investigate a possible mediating effect of coping strategies on the relationship between personality traits and change in blood pressure. Extraversion, agreeableness, openness, and problem-solving skills were identified as possible protective factors against cardiovascular risk, confirming previous research in this regard. However, the effect of these was different for gender and ethnic subgroups. Preconditions for a possible mediation role for coping in the relationship between personality and change in blood pressure were not met. Future research should further explore gender and ethnic differences in the relationship between personality, coping, and cardiovascular health.

## Introduction

A World Health Organization study reported South Africa has the highest prevalence of hypertension in adults over the age of 50 compared to China, Ghana, India, Mexico, and the Russian Federation.^1,2^ In response to this reality, the African Prospective Study on the Early Detection and Identification of Cardiovascular Disease and Hypertension (African-PREDICT) aims to identify predictors/early markers of cardiovascular disease amidst apparently healthy, normotensive young adults in the North-West Province, South Africa.^3^ The study has a longitudinal design, placing a focus on this population to investigate the development of cardiovascular conditions over time. Many of the active sub-studies under the African-PREDICT study focus on physiological predictive factors,^3^ yet research suggests that these do not explain the entirety of risk factors associated with the development of hypertension.^4^ Previous research has found that psychological factors such as coping with stress and personality traits may put individuals at risk of developing cardiovascular conditions related to hypertension.^4–7^ This paper reports on an African-PREDICT sub-study, building on previous research suggesting that personality traits relate to the selection of coping strategies,^8^ which in turn can influence how the body reacts to stress and the effects thereof on blood pressure.^4,9^

Hypertension is the diagnosis made when a person is subject to repeated, reproducible measures of elevated blood pressure.^10^ Research has found a close relationship between the mechanisms underlying blood pressure and the experience of stress.^4,11^ Stress is both the psychological perception of not having the resources necessary to meet the demands of a situation and the body's physiological response to that experience.^12^^–^^14^ Physiologically, after a stressful event has occurred, the one component of the autonomic nervous system (ANS), the parasympathetic system, counteracts the other component, the sympathetic nervous system, to restore the body to a restful state.^15^ The ANS also regulates mechanisms underlying blood pressure.^15–16^ This includes, among other factors, cardiac output (blood volume the heart pumps per minute) and peripheral resistance (level of arterial resistance to heart-pumped blood).^17,18^ Crucially important to the experience of stress is how individuals respond to or cope with stress.^19^ Various coping researchers agree that the study of coping is fundamental to understanding how stress affects people over the long term and its influence on the development of physical health or disease.^9^ The current study aligns with the three strategies of Amirkhan,^20^ namely (i) problem-solving: approaching the stress-inducing situation to solve the related problem; (ii) avoidance: emotional responses to stress that involve forms of withdrawal from the problem, and (iii) seeking support: actively turning to others for comfort, help and advice. Literature acknowledges that personality plays a role in how people select strategies to cope with stress.^21,22^

Personality refers to a person's general and stable patterns of thoughts, emotions, and behavior.^22^ Previous literature has found that personality traits influence the way stress is perceived, as well as one's selection of coping strategies.^22^ The five-factor theory is one of the more extensively researched and evidence-based personality theories found to relate to the selection of coping strategies.^21,23,24^ It categorizes five groupings of personality traits, namely (i) extraversion, the tendency excitement-seeking, liveliness and positive affectivity, (ii) neuroticism, found in those with high scores in depression, anxiety, affective instability and self-consciousness, (iii) conscientiousness, which refers to effort, order, prudence, self-discipline and dutifulness, (iv) openness to experience, characterized by aesthetics, ideas, actions, values and imagination, and (v) agreeableness, manifested by straightforwardness, compliance, tender-mindedness, prosocial tendencies and modesty.^25^ A meta-analysis by Connor-Smith and Flachsbart^23^ investigated possible relations between the five-factor personality traits and coping strategies. Personality was weakly related to broad coping, yet each of the five traits predicted specific coping strategies.^23^

Previous studies^5,6^ have turned toward investigating trait outcomes, or behaviors associated with traits, but still only aimed to find associations with hypertension, rather than predictive values. Terracciano et al,^7^ however, used the five-factor personality traits to predict blood pressure patterns 7 years later. In addition, Lu and Wang^26^ and Jonassaint et al^27^ also reported prospective cardiovascular health benefits and risks of personality traits in their association with adaptive physiological reactivity to recurrent social stressors. Psychological factors, including personality traits and coping with stress could be associated with a significant change in blood pressure over time.^4^^,^^7^ This association has, however, not been comprehensively assessed within the South African context, and specifically not in a normotensive baseline sample of young adults. The following question thus arises: what is the association between personality traits, coping strategies and change in blood pressure in young adults in the African-PREDICT study over the course of 5 years?

## Aims

The aims of this study were to determine if:

1. there is a statistically significant association between personality traits, coping strategies, and 5-year change in blood pressure of healthy South African young adults
2. coping strategies have a significant mediating effect on the relationship between personality traits and 5-year change in blood pressure of healthy South African young adults

## Methodology

The current study is part of a larger longitudinal study which has been described in various previous literature,^3,28–29^ only information relevant to the current sub-study is discussed.

### Participants

South African male and female young adults (20-30 years) of black and white ethnicity were recruited via advertisements, workplace access, and field workers. Inclusion criteria were apparent health and normotensive status (clinical brachial blood pressure of <140/90 mm Hg).^3^ Exclusion criteria were not residing in or around the Potchefstroom area, a positive HIV status, a chronic disease diagnosis or medication, phobia for needles, fever, breastfeeding or pregnant, and not English proficient (speaking and reading/writing).^3^

The total African-PREDICT sample includes 1202 participants at the beginning of the study. Not all follow-up data were collected at the time of data analysis for this sub-study; therefore, only the 105 participants who had complete data sets for baseline and 5-year follow-up ambulatory blood pressure readings, as well as the two psychological measurements, were included. The number of participants (*n*) and mean age according to sex and ethnic grouping at baseline and at 5-year follow-up are displayed in Table 1. The participants were predominantly female (61.9%) and black (61.9%). At baseline, the mean age of the participants was 24.93 years (black = 24.34 years; White = 25.9 years; male = 24.63; female = 25.12).

**Table 1.** Demographic Characteristics.

	Black (*n*)	White (*n*)	Total
**Male (*n*)**	24 *(22.8%)*	16 *(15.2%)*	40 *(38.0%)*
1	23.83	25.81	24.63
2	28.83	30.81	29.63
**Female (*n*)**	41 *(39.0%)*	24 *(22.8%)*	65 *(61.9%)*
1	24.63	25.96	25.12
2	29.63	30.96	30.12
**Total**	65 *(61.9%)*	40 *(38.0%)*	**105 *(100%)***
1	24.34	25.9	24.93
2	29.34	30.9	29.93

*Note.* 1 = Mean age at baseline; 2 = Mean age at 5-year follow-up.

### Measures and Data Collection

## 24-Hour Ambulatory Blood Pressure (AP) and ECG Monitoring

Twenty-four-hour ambulatory blood pressure measurements (ABPM) were determined both at baseline and 5-year follow-up with a Card(X)plore device validated by the British Hypertension Society (Meditech, Budapest, Hungary). A fitted cuff was used on the participant's nondominant arm that measured blood pressure in 30-minute intervals during daytime (08:00-22:00) and hourly during night-time (22:00-06:00). Change in blood pressure was calculated as BP (follow-up) - BP (baseline) / BP (baseline).

### Basic Traits Inventory Short

The Basic Traits Inventory (BTI)^25^ was developed specifically for use in the South African context. It is based on the five-factor model (FFM) structure of personality consisting of five global domains, namely extraversion, neuroticism, contentiousness, openness, and agreeableness.^24^ This study used the shortened version of the BTI (BTI-Short). Each item relates to a personality trait.^24^ The BTI-short was found to be valid for cross-cultural studies in black South African language groups.^24^ The reliability of this scale was also calculated to be acceptable for this study (see Table 2).

**Table 2.** Mean Personality and Coping Scores by Sex and Ethnicity.

		**Mean scores**		
	Black (*n *= 65)	White (*n *= 40)	Total (*n *= 105)	Cronbach's α
**Personality Traits**				
Extroversion	3.85	3.77	3.82	.80
*Male*	3.71	3.80	3.75	
*Female*	3.94	3.75	3.87	
Neuroticism	2.51	2.33	2.44	.79
*Male*	2.34	2.15	2.26	
*Female*	2.61	2.44	2.55	
Conscientiousness	4.02	3.91	3.98	.81
*Male*	4.01	3.82	3.93	
*Female*	4.03	9.98	4.01	
Openness	4.14	3.88	4.07	.77
*Male*	4.11	4.02	4.10	
*Female*	4.17	3.78	4.03	
Agreeableness	4.29	3.94	4.15	.75
*Male*	4.12	3.86	4.02	
*Female*	4.38	3.99	4.24	
**Coping Strategies**				
Problem-Solving	1.47	1.55	1.50	.86
*Male*	1.58	1.52	1.56	
*Female*	1.40	1.57	1.47	
Avoidance	2.01	2.23	2.09	.78
*Male*	2.07	2.31	2.17	
*Female*	1.79	2.19	2.05	
Support Seeking	1.76	1.80	1.78	.83
*Male*	1.93	1.83	1.89	
*Female*	1.66	1.78	1.71	

### Coping Strategy Indicator

The coping strategy indicator (CSI)^20^ measures three fundamental coping strategies through 11 items each (total 33), namely problem-solving, seeking social support, and avoidance. Respondents are required to recall an important or significant stressful event from the last 6 months and briefly describe it.^20^ Amirkhan^20^ reported good internal consistency with Cronbach's alpha coefficients ranging between .839 and .928; as well as good test-retest reliability with Pearson coefficients ranging between .80 and .86. The reliability of this scale was also calculated to be acceptable in this study (see Table 2).

### Data Analysis

Descriptive statistics were calculated for age, sex, ethnicity, and all items in the BTI-Short and CSI, as well as change in blood pressure from baseline to follow-up. Cronbach's alpha was also calculated to establish the internal consistency of these measures.

Spearman's rank order correlations (due to the non-parametric quality of the sample) were calculated to determine relationships between changes in blood pressure, personality and coping. Factorial (two-way) ANOVAs were calculated to determine the main and interaction effects of (i) sex and personality on change in blood pressure, (ii) ethnicity and personality on change in blood pressure, (iii) and sex and coping on change in blood pressure, and (iv) ethnicity and coping on change in blood pressure.

**Figure 1.**
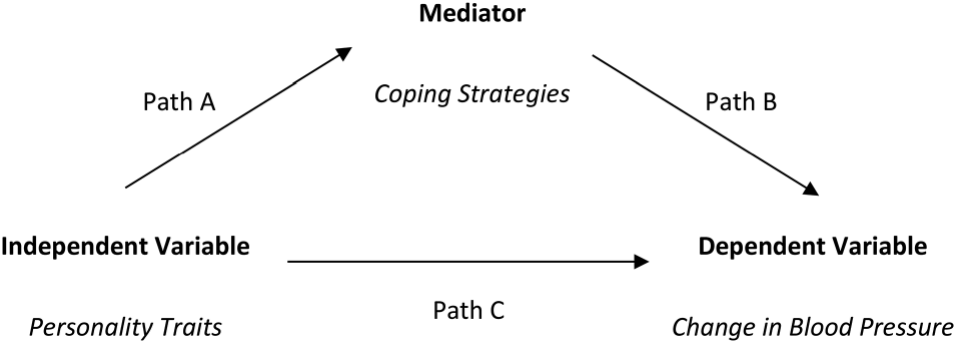
Conditions for mediation.

To determine whether coping mediates between personality and change in blood pressure, Baron and Kenny's^30^ suggestion was followed by testing three conditions (see Figure 1), (i) the association between the independent variable (personality trait) and the mediator (coping strategy) (path A), (ii) the association between the mediator (coping strategy) and the dependent variable (change in blood pressure) (path B), and (iii) the association between the independent variable (personality trait) and the dependent variable (change in blood pressure) (path C) when the effect of the mediator (coping strategy) (path B) is controlled.^30^

As per the study aims, tests were two-tailed with the type I error rate (α=0.05) . Effect sizes for determining the practical significance of correlations are indicated by *r* with 0.1 ≤ *r* < 0.3 (small); 0.3 ≤ *r* < 0.5 (medium); and *r* ≥ 0.5 (large). Partial Eta Squared effect sizes were calculated for factorial ANOVAS where, *η*_p_^2^ = 0.01 (small); *η*_p_^2^ = 0.06 (medium); and, *η*_p_^2^ ≥ 0.14 (large).^31^ Due to the relatively small sample size, only medium or large effect sizes are reported and interpreted.

### Ethical Considerations

The larger African-PREDICT study followed guidelines of the 2008-revised Helsinki Declaration of 1975 for conducting research on human participants.^3^ Ethical approval was undersigned by both the South African National Department of Health and the Health Research Ethics Committee (HREC) of the North-West University, with ethics approval number for the larger study, NWU-00001-12-A1 and for this sub-study, NWU-02073-20-A1.

## Results

Table 2 shows the mean personality and coping scores for the total group, and sex and ethnicity subgroups. Cronbach's alpha for half of the psychological constructs were good (*α* ≥ 0.8), while the rest were still deemed acceptable (*α* ≥ 0.7).^32^

Table 3 shows the mean blood pressure readings of the total group and each subgroup at baseline, 5-year follow-up, and change in blood pressure over the 5 years. The mean change in SBP for the total group was 8.09, and for DBP was 7.47.

**Table 3.** Mean Blood Pressure at Baseline, Follow-up and Change.

	Baseline			Follow-up			Change		
Systolic BP									
	B	W	Total	B	W	Total	B	W	Total
	116.11	112.44	114.71	124.55	119.96	122.80	8.44	7.53	8.09
M	121.40	124.11	122.48	131.39	132.14	131.69	9.99	8.03	9.21
F	113.02	104.66	109.93	120.55	111.84	117.33	7.53	7.19	7.40
Diastolic BP									
	B	W	Total	B	W	Total	B	W	Total
	77.81	73.33	76.10	86.05	79.55	83.57	8.24	6.22	7.47
M	79.13	77.39	78.43	88.45	83.89	86.63	9.32	6.50	8.19
F	77.04	70.63	74.67	84.65	76.66	81.70	7.60	6.03	7.02

*Note.* B = Black; W = White; M = Male; F = Female.

Table 4 shows Spearman's rank order correlations (*ρ*) between changes in blood pressure, personality, and coping. Due to the large number of variables and subgroups, the table is not structured according to standard correlation matrix guidelines, rather, an adjusted structure is presented to show all relevant information.

**Table 4.** Correlation Matrix with Spearman's Rank Order Correlations.

	SBP change			DBP change			Problem Sol.			Avoidance			Social Supp.		
	B	W	T	B	W	T	B	W	T	B	W	T	B	W	T
Extraversion	−0.233	0.012	−0.140	−0.179	−0.009	−0.096	−0.040	−.362*	−0.167	−0.085	−0.037	−0.103	0.024	0.156	0.078
*Male*	−0.296	0.391	−0.047	−.478*	0.428	−0.174	0.136	−0.130	0.042	−0.202	0.235	−0.033	0.192	0.168	0.154
*Female*	−0.117	−0.230	−0.175	0.031	−0.277	−0.056	−0155	−.517**	−.300*	−0.003	−0.181	−0.136	−0.007	0.116	0.057
Neuroticism	0.027	0.014	0.014	0.065	−0.071	0.026	−0.052	0.187	0.036	−.298*	−.315*	−.278**	−0.091	−0.153	−0.122
*Male*	0.066	−0.204	−0.039	−0.169	−0.200	−0.154	−0.084	0.107	−0.011	−0.171	−0.321	−0.251	−0.128	−0.324	−0.199
*Female*	0.029	0.121	0.056	0.198	0.056	0.150	0.007	0.266	0.084	−.337*	−0.277	−.264*	−0.043	−0.060	−0.057
Conscient.	−0.036	−0.124	−0.054	−0.064	−0.009	−0.029	−0.177	−0.262	−.208*	−0.064	−0.011	−0.056	−0.070	0.041	−0.022
*Male*	0.128	0.184	0.204	−0.199	0.423	0.090	−0.312	−0.074	−0.218	−0.272	0.289	−0.020	−0.117	0.230	0.043
*Female*	−0.139	−0.337	−0.194	0.020	−0.295	−0.087	−0.107	−0.362	−0.199	0.033	−0.208	−0.067	−0.045	−0.070	−0.042
Openness	0.004	−0.236	−0.089	−0.101	−0.097	−0.076	−0.063	−0.042	−0.065	−0.193	−0.147	−.233*	0.012	0.223	0.086
*Male*	−0.022	0.048	−0.006	−0.214	0.202	−0.091	−0.077	0.105	−0.020	0.269	−0.035	0.078	0.071	0.127	0.085
*Female*	−0.005	−.470*	−0.171	0.016	−.411*	−0.097	−0.058	−0.093	−0.103	−.423**	−0.244	−.418**	0.036	0.277	0.104
Agreeablen.	−0.159	−0.077	−0.118	−0.240	−0.188	−0.180	−0.092	−.407**	−.218*	−0.147	−0.059	−0.164	−0.079	−0.047	−0.072
*Male*	−0.175	0.312	0.005	−.517**	0.054	−0.207	−0.261	−0.430	−.317*	0.036	0.063	−0.015	−0.093	−0.064	−0.051
*Female*	−0.066	−0.294	−0.118	−0.017	−0.374	−0.119	0.067	−0.320	−0.120	−0.205	−0.177	−0.222	−0.007	−0.071	−0.034
Problem Sol.	0.138	−0.140	0.040	0.203	0.102	0.166									
*Male*	−.405*	−0.464	−.344*	0.178	−0.061	0.123									
*Female*	.430**	0.067	.295*	0.220	0.189	0.202									
Avoidance	0.050	0.206	0.119	−0.066	0.126	−0.013									
*Male*	0.166	0.414	0.276	−0.099	0.325	0.049									
*Female*	−0.011	−0.030	0.002	−0.069	−0.040	−0.073									
Social Supp.	−0.026	0.024	−0.015	−0.019	−0.073	−0.052									
*Male*	−0.153	0.381	0.087	0.035	0.147	0.091									
*Female*	−0.038	−0.240	−0.133	−0.091	−0.158	−0.148									

Note.* p < .05; ** p < .01; B = Black; W = White; T = Total; Conscient. = Conscientiousness; Agreeablen. = Agreeableness; Problem Sol. = Problem-Solving; Social Sup. = Seeking Social Support.

### Path B: Coping Strategies and Change in Blood Pressure

A significant negative correlation with medium effect size was found between problem-solving coping and change in SBP for the total male group (*ρ* = −.344; *p *< .05) and the black male group (*ρ* = −.405; *p *< .05). A significant positive correlation was found between problem-solving and change in SBP with a medium effect size for the black female group (*ρ* = .430; *p *< .001).

#### Coping as Mediator Between Personality and Change in Blood Pressure

It is clear from the correlations that although extraversion, agreeableness and openness show significant, medium and large effect size correlations with coping and change in blood pressure, none of these were found for the total group or in similar variable clusters in any of the subgroups. The only possibility would have been problem-solving as a mediator between extraversion or agreeableness and change in blood pressure, however, problem-solving was associated with change in SBP while extraversion and agreeableness were associated with change in DBP, and these were found in different participant subgroups. However, as differences were noted in how personality and coping were associated with blood pressure according to sex and ethnicity, interaction (moderation) effects were further explored.

### Interaction Effects

Only practically significant moderation effects for statistically significant correlations are reported and interpreted. A significant interaction effect with large effect was found for sex and problem-solving coping on change in SBP with *F* (1, 101) = 17.331. *p *< .01, *η*_p_^2 ^= .15 (see Table 5).

**Table 5.** Interaction Effect of Sex and Problem-Solving Coping on SBP.

Source	Type III Sum of Squares	df	Mean Square	*F*	Sig.	Partial Eta Squared
Corrected Model	667.079^a^	3	222.360	6.617	0.000	0.164
Intercept	7052.552	1	7052.552	209.861	0.000	0.675
Sex	78.373	1	78.373	2.332	0.130	0.023
NProblem	12.639	1	12.639	0.376	0.541	0.004
sex * NProblem	581.746	1	581.746	17.311	0.000	0.146
Error	3394.187	101	33.606			
Total	10934.125	105				
Corrected Total	4061.265	104				

*Note.* a. R Squared = .164 (Adjusted R Squared = .139).

**Figure 2.**
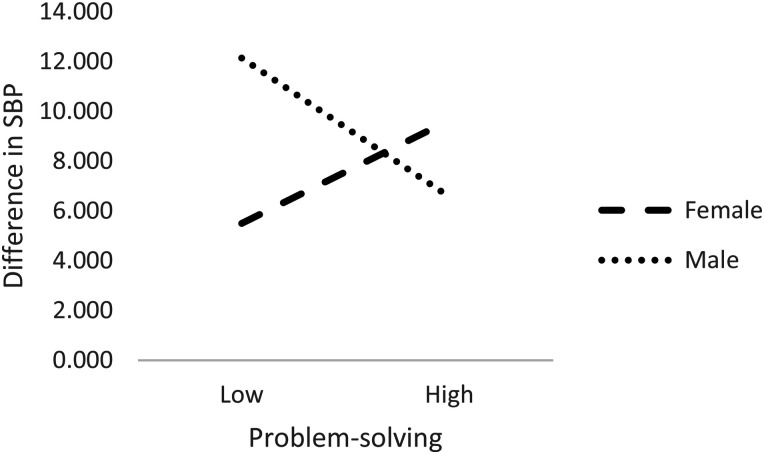
Graphic demonstration of interaction effect: sex and problem-solving on SBP.

Based on this result, Figure 2 demonstrates how men low in problem-solving (*x* = 12.13) show a significantly larger increase in SBP than women low in problem-solving (*x* = 5.49).

## Discussion

### The Relationship Between Personality Traits and Coping Strategies

Both extraversion (in white women) and agreeableness (in the white sample and men) were negatively correlated with problem-solving, while openness (in females and black females) was negatively correlated with avoidance. The negative correlation between extraversion and problem-solving is a surprise, as one would expect that characteristics of extraversion (especially optimism and assertiveness) would facilitate problem-solving behavior.^23^^,^^33^ For this sample, in contrast to previous literature,^23^ the opposite was found to be true for white females, and as the reason for this discrepancy and specific subgroup is not apparent from the current results, it could be further explored in future research.

The significant negative correlation between agreeableness and problem-solving for the white and the male group is also a surprise, compared to previous research (Leszko et al;^21^ Connor-Smith and Flachbart^23^). However, from a theoretical viewpoint, this make sense, as agreeableness is characterized by being trustworthy, friendly, considerate, generous, helpful, and altruistic.^33^ These characteristics might imply that agreeable persons are more focused on interpersonal harmony than setting challenging goals for themselves (such as solving problems when experiencing stress).^34^ Therefore, given their need for strong social networks, agreeableness would rather be expected to correlate with seeking social support, as was found by Leszko et al.^21^

The significant negative correlation between openness and avoidance for the female group and the black female group is also not unexpected, as openness is characterized by being creative, intellectual, imaginative, curious, cultured, and complex^33^ characteristics that one would expect to foster engagement rather than avoidance, as implied by previous research.^21,23^

### The Relationship Between Personality and Change in Blood Pressure

For black males in this sample, significant negative correlations were found between extraversion and change in DBP (medium effect size) and between agreeableness and change in DBP (large effect). For white women in this sample, a significant negative correlation was found between openness and change in both DBP and SBP (with medium effect sizes). Higher extraversion, agreeableness and openness are therefore related to lower changes in BP for black men and white women, respectively.

Theoretically, it makes sense for extraversion to negatively correlate with change in blood pressure, as many of its qualities like being warm, sociable, excitement-seeking, active, spontaneous, and optimistic are associated with being more likely to focus on the positive aspects of a stressor.^35^ In support of this, Lu and Wang^26^ found in two different studies that participants scoring high in extroversion exhibit more adaptive physiological reactivity to recurrent social stressors, which may be beneficial to their health.

Another study found that individuals scoring low on extraversion had a more elevated resting heart rate as compared to those scoring higher on extraversion.^36^ They subsequently hypothesized that this may lead to those lower in extraversion being at greater cardiovascular risk.^36^ Jonassaint et al^27^ found higher extraversion to be a protective factor due to its association with lower cardiovascular reactivity. The studies above seem to support the notion of extraversion being a protective factor to cardiovascular health but do not help to explain why this effect was found in black men only in this study.

The negative correlation between agreeableness and change in DBP (with large practical value) relates to previous research which found agreeableness to be a protective factor against cardiovascular risk factors, however, with regards to SBP and not DBP.^7,37^ Terracciano et al^7^ and Vecsey-Nagy et al^37^ found agreeableness to be associated with lower night-time SBP, while Sutin et al^38^ found low agreeableness to relate to the risk of arterial thickening. This is a risk factor for high blood pressure, as it causes a more strenuous peripheral resistance when blood is circulated through the veins.^17,18,39^ Both Terracciano et al^7^ and Sutin et al^38^ studied the Italian population (*n* = 2848 and *n* = 5614, respectively) and had a prospective angle by taking measures over time (7 years later and concurrently over 3 years, respectively). The sociable and harmonious characteristics of those high in agreeableness could also indicate a lifestyle that is focused on cooperative achievements rather than on (stress-inducing) high-stakes personal challenges.^34^ The implication of this is not necessarily how those high in agreeableness cope with stress, but rather how they do not position themselves in scenarios likely to bring about stress. When combining the longitudinal studies^7,38^ with the current results, it seems as though there are grounds for investigating agreeableness as a protective factor against cardiovascular disease development.

Reasons why extraversion and agreeableness could be protective factors in black males specifically may be found in Langa^40^ on black masculinity. According to Langa,^40^ assertion of dominance is particularly important to black masculinity, implying that those who do not possess the personal resources to assert dominance could be subject to higher levels of social stress. Therefore, having a personal resource such as extraversion (being assertive, sociable, and optimistic)^33^ could be beneficial for particular black males in navigating these social pressures. Characteristics of *agreeableness* like being trustworthy, friendly, considerate, and being more focused on interpersonal harmony^33,^^34^ could also serve the purpose of creating a social status less likely to be challenged.

For the white female group, negative associations with medium effect sizes were found between openness and both change in SBP and DBP. Openness, characterized by being creative, intellectual, imaginative, curious, cultured, and complex^33^ therefore seems to be a protective factor for white women in this sample. This notion is supported by research on a female sample that found that those higher in openness have a more adaptive cardiovascular stress response profile in the context of changing acute stress exposures.^41^ Lü et al^42^ also found in a group of female participants that higher openness was associated with less SBP reactivity to a stressor while it was also associated with greater SBP and DBP adaptation with greater decreases in blood pressure reactivity across two successive stress exposures. In Dermody et al,^43^ openness emerged as an independent negative predictor for cardiometabolic risk. Theoretically, this could make sense seeing that openness is associated with practicing reflection and assessing personal performance.^34^ In turn, this leads to more personal growth opportunities, which is related to more positive functioning.^44^

To further explore possible reasons why the above correlations were only found in ethnic-specific subgroups, we can consider that according to Allik and McCrae,^45^ differences in personality between South African ethnic groups are related more to the different ways in which these personality traits are expressed from an individualist or collectivist cultural viewpoint.^45^

### The Relationship Between Coping Strategies and Change in Blood Pressure

A significant negative correlation was found between problem-solving and change in SBP for both men and black men; thus, for those higher in problem-solving coping, the change in SBP was significantly less. This finding is perhaps no surprise, as problem-solving coping refers to approaching stress-inducing situations directly,^20^ and in doing so, eliminating the potential for a continuous experience of stress. This emphasizes the importance of effective coping strategies for general health, which is supported by Lindquist,^46^ who found that in men, occupational stress correlated with avoidance coping, and Shafiq et al,^47^ who found that problem-solving coping predicted better quality of life in patients with blood pressure ailments. Reasons for this correlation being found in men and not women are not clear from this study. However, the reason for this correlation specifically in black men could perhaps also be linked to black masculinity,^40^ as a direct problem-solving approach could benefit the expectation to assert dominance.

For black women, the opposite correlation was found between problem-solving and SBP, implying that problem-solving is not considered a potential protective factor against change in blood pressure for this subgroup and could potentially be a risk factor. In support of these contrasting correlations, the significant and practically large interaction effect found for sex and problem-solving coping on change in SBP clearly suggests that the different correlations found in this sample are related to sex differences. In this sample of young adults, problem-solving is more beneficial for men, as compared to women, in protection against change in blood pressure over 5 years. Reasons for this is not clear in this study but could provide direction for future research.

## Conclusion

Before a final conclusion can be made, some limitations of the study must be considered. First, unfortunately, only data from a relatively small sample was available for this study, and even though this was controlled for by using non-parametric statistics, one should be careful not to generalize these findings to the young adult South African population. Secondly, as requirements for mediation were not met, both aims were not comprehensively addressed.

Preconditions for possible mediation were not met, showing that, at least in this sample, coping does not act as a mediator. The surprising finding that extraversion and agreeableness negatively correlated with problem-solving*,* might explain why the mediation requirements were not met, but it could also imply that the relationship between personality and coping is more complex than often described in literature and not always the same for all sex and ethnic groups.

Different results for subgroups could direct future research, especially future substudies of the African-PREDICT study. Based on the ethnic-specific correlations found for personality traits (especially extraversion) with the other variables, we propose that future research follow the suggestions of Allik and McCrae,^45^ and Weisberg et al^48^ to consider the ways in which this personality trait is expressed from an individualist or collectivist cultural viewpoint and the role that personality traits play in these contexts.
